# Chitosan-Based Polymer Blends for Drug Delivery Systems

**DOI:** 10.3390/polym15092028

**Published:** 2023-04-25

**Authors:** Malkiet Kaur, Ameya Sharma, Vivek Puri, Geeta Aggarwal, Paramjot Maman, Kampanart Huanbutta, Manju Nagpal, Tanikan Sangnim

**Affiliations:** 1 Chitkara College of Pharmacy, Chitkara University, Punjab 140401, India; 2 Chitkara School of Pharmacy, Chitkara University, Himachal Pradesh 174103, India; 3 Department of Pharmaceutical Sciences, Delhi Pharmaceutical Sciences and Research University, New Delhi 110017, India; 4 Paraxel International Pvt Ltd., Mohali 140308, India; 5 School of Pharmacy, Eastern Asia University, Pathum Thani 12110, Thailand; 6 Faculty of Pharmaceutical Sciences, Burapha University, Chonburi 20131, Thailand

**Keywords:** biopolymers, polymer blends, chitosan, drug delivery, nanocarriers

## Abstract

Polymers have been widely used for the development of drug delivery systems accommodating the regulated release of therapeutic agents in consistent doses over a long period, cyclic dosing, and the adjustable release of both hydrophobic and hydrophilic drugs. Nowadays, polymer blends are increasingly employed in drug development as they generate more promising results when compared to those of homopolymers. This review article describes the recent research efforts focusing on the utilization of chitosan blends with other polymers in an attempt to enhance the properties of chitosan. Furthermore, the various applications of chitosan blends in drug delivery are thoroughly discussed herein. The literature from the past ten years was collected using various search engines such as ScienceDirect, J-Gate, Google Scholar, PubMed, and research data were compiled according to the various novel carrier systems. Nanocarriers made from chitosan and chitosan derivatives have a positive surface charge, which allows for control of the rate, duration, and location of drug release in the body, and can increase the safety and efficacy of the delivery system. Recently developed nanocarriers using chitosan blends have been shown to be cost-effective, more efficacious, and prolonged release carriers that can be incorporated into suitable dosage forms.

## 1. Introduction

Since the 1980s, researchers have been working on polymeric drug delivery systems or devices that could be safely introduced/inserted into the body. Polymeric materials are being used in a variety of biomedical applications, including drug delivery systems, the implantation of medical devices, artificial organs, tissue engineering scaffolds, ophthalmology, prosthetics, bone healing, dentistry, and other applications [1,2]. The polymers used in the drug delivery systems are classified based on their characteristics (Table 1) [3,4]. Polymers can be used to construct polymeric nanocarrier systems that improve drug bioavailability by enhancing their retention time, lowering their adverse effects, enhancing drug solubility, and reducing the required dosing frequency [5,6]. Natural polymers, which are essentially polysaccharides, play a vital role in controlling the drug rate, managing dosage forms, and enhancing the physicochemical parameters. Because of their low cost, biodegradability, biocompatible structures, availability, and safety, polymers are widely used as excipients in the pharmaceutical sector [7,8]. Even though these polymers are readily available, there is always a need for new and enhanced materials. The substantial safety testing required for new materials is frequently a limiting factor for their application in new therapeutic products, even though it is possible to synthesize new polymers in order to achieve the desired functionality. The introduction and use of novel polymers have been severely constrained owing to the substantial safety testing necessary for new excipient licensing. The limitations of individual polymers can be overcome through a combination of existing permitted polymers, which is a viable option. There are several ways to alter the properties of a polymer, such as curing, blending, grafting, and derivatization [9]. Blending is the process of physically mixing two or more polymers to produce a polymer with specified properties [10,11,12]. Graft copolymerization is the process of covalently bonding one polymer chain to another; it has no time restriction, as it can take place in a matter of seconds, minutes, or even hours. Curing involves the polymerization of an oligomer mixture to create a coating that adheres to the substrate through physical forces. It is a very quick procedure that takes only a few seconds. Derivatization involves replacing reactive groups on the polymer chain with simple molecules, e.g., etherification and esterification.

**Figure 1:**
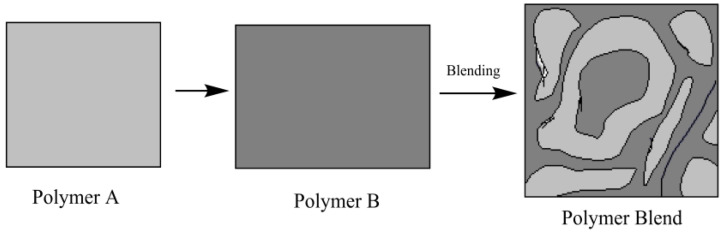
Hypothetical scheme of the preparation of a polymer blend.

Polymer blending method offers a low-cost process, and instead of a complicated chemical process, simple physical processes are involved in it. The hypothetical blending of polymers is shown in Figure 1. Thus, polymer blends possess an alternative approach to solve numerous formulation and drug delivery difficulties, especially in the context of the time and the resources needed in order to secure a regulatory approval every time a new excipient is to be used [13,14]. After reviewing approximately 13,000 reports from the Science Direct, PubMed-like research engines on chitosan-based drug delivery systems, it was observed that blending polymer exhibits several characteristics of the polymers, such as controlling the rate, duration, and location of drug release in the body, and can increase the safety and efficacy of the delivery system [15]. At the nanoscale, these potential carriers can carry bioactive substances to targeted cells and tissues by causing a minimal immunological response [16].

**Figure 2:**
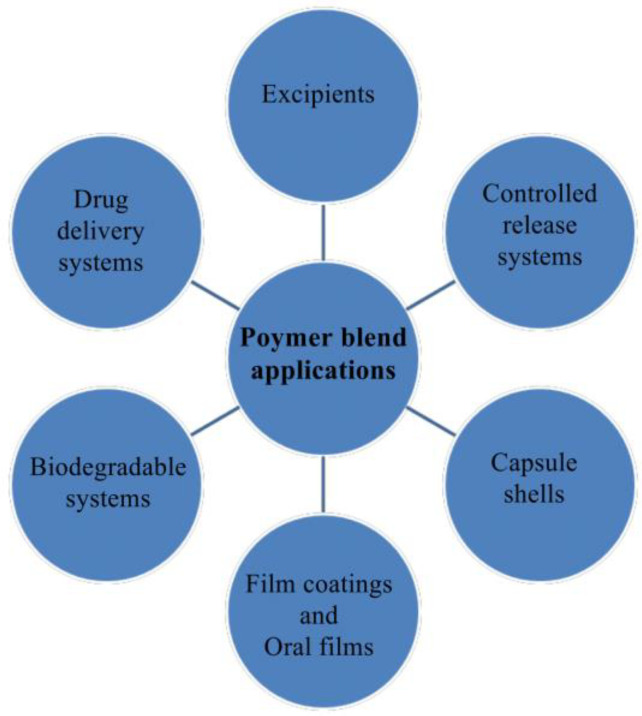
Applications of polymer blends.

There are various types of polymer blends, including thermoplastic–thermoplastic, rubber–thermoplastic, thermoplastic–thermosetting, rubber–thermosetting, and polymer filler blends [17,18]. Polymer blending has various applications (Figure 2) [14,19]. Polymer blends can be classified into heterogeneous (miscible on a molecular level) and homogeneous (immiscible on a molecular level) blends. If the combining of the polymers is exothermic, then the system is driven toward miscibility. However, the combining of polymers is only exothermic when strong; specific interactions occur between the polymers involved [20]. Hydrogen bonding, ionic interactions, and dipole–dipole interactions are the most prevalent interactions observed in polymer blends. Fourier transform infrared (FTIR) spectroscopy, ellipsometry, small-angle neutron scattering, nuclear magnetic resonance spectroscopy, and neutron reflectivity are some of the techniques that can be used to investigate the specific interactions occurring between polymers [17].

Chitosan, a natural polymer, has attracted great interest from researchers owing to its unique properties, such as biocompatibility, biodegradability, and non-toxicity [21,22]. In this article, the authors have mainly focused on chitosan-based polymer blends in drug delivery [23]. It is widely used as a carrier in drug delivery, orthopedics, cell delivery systems, wound healing, bone healing, and ophthalmology as it can increase the functioning of macrophages and polymorphonuclear cells and can enhance the fibroblast proliferation and migration [24].

## 2. Overview of Chitosan

**Figure 3:**
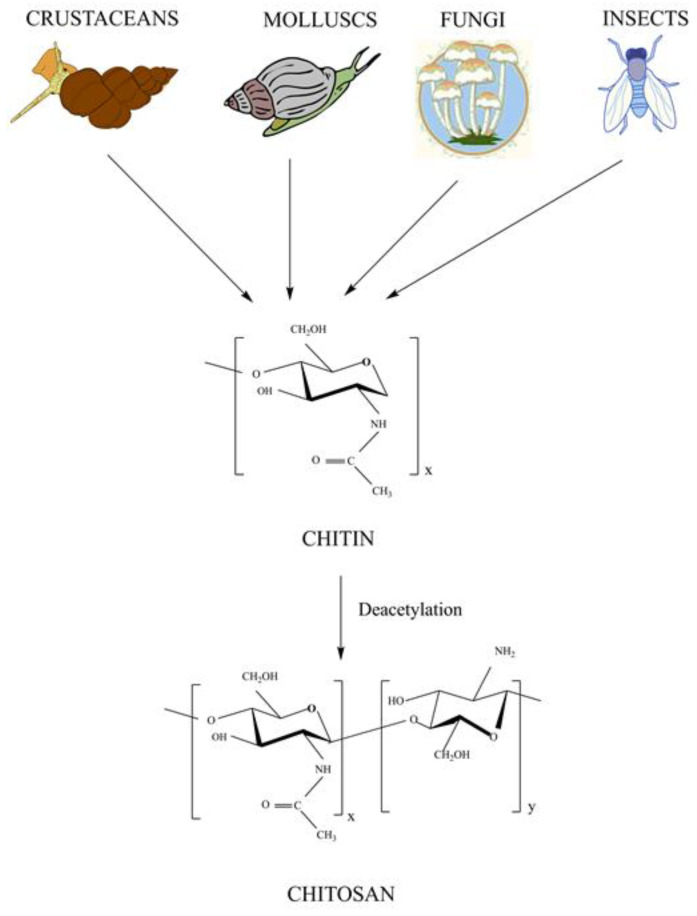
Fabrication of chitosan.

Chitosan is a generic name for a linear random copolymer of β-(1-4) linkedD-glucosamine, with a linear backbone joined by glycoside linkage in its chemical structure. It is a natural carbohydrate and a cationic polymer obtained from the de-acetylation of chitin (shown in Figure 3). Chitosan is gaining the interest of researchers due to the presence of amine at C-2 position of glucosamine residues. This large concentration of amines gives chitosan key functional characteristics. Chitosan exhibits antimicrobial activity and hemostatic and clotting capabilities [25]. Insects (cuticle, beetle cocoon, and ovipositors), crustaceans (shrimp shell and crab shell), squids (*Loligo* stomach and *Ommastrephes* pen), centric diatoms (algae and *Thalassiosira fluviatilis*), and fungi (*Aspergillus nidulans* and *Mucor rouxii*) are the main sources of chitin. Chitosan’s molecular weight and level of deacetylation affects physico-mechanical properties. The chitosan, having a higher degree of deacetylation, shows toxicity according to their molecular weights, i.e., are less toxic for low molecular weight and highly toxic for high molecular weights. However, the chitosan having a lower degree of deacetylation acts as an absorption enhancer at both high and low molecular weights. The solubility and degradation of chitosan are influenced by its molecular weight. The high molecular weight of chitosan is less soluble and degrades more slowly than lower molecular weight chitosan [26,27].

It can also act as a fat attractor by attaching to dietary lipids. In most physiological fluids, the basic amine groups of these polysaccharides are protonated and can become positively charged [28]. Chitosan has a pH-dependent behavior in solution, as well as several interesting biopharmaceutical features (such as mucoadhesiveness and the ability to open epithelial tight junctions) [29]. It can also exhibit gel-forming properties at a low pH [30]. It has gained the significant interest in biomedical and pharmaceutical applications as a versatile biopolymer. Chitosan can be modified using the hydroxyl and amine group on chitosan (a site for modification). Due to the protonation of the amino groups, it has positive charges in acidic media and can connect with the mucin’s negatively charged residues to enhance the mucoadhesive characteristics. Chitosan’s antibacterial properties can be explained by two different methods [31]. According to the first theory, the positive charges on chitosan could interact with the negative charges on bacterial cell walls to change permeability and cause solute leakage. The second suggested that it might interact with bacterial DNA cells to prevent the synthesis of RNA. Chitosan’s polycationic nature also makes it possible to explain that it has analgesic properties. In fact, the D-glucosamine, residues of amino groups can protonate in the presence of proton ions generated during inflammation, having an analgesic effect [32]. Chitosan blends exhibiting controlled release properties are discussed in the below section and its applications in various drug delivery systems are discussed in Section 3.

### Research Reports on Chitosan Blends with Other Polymers

Some properties of chitosan can be modified by blending it with other natural and synthetic polymers such as zein, curdlan, sodium alginate, konjac, polylactic acid, glucomannan, polycaprolactone, poly(ethylene oxide), graphene oxide, and poly(vinyl pyrollidine) [33,34]. The existence of -NH_2_ and -OH groups in chitosan allows them to interact with other polymers and biological molecules. Chitosan, alone or in combination, is a suitable substrate for developing various nanocarriers such as films, hydrogels, porous foams, beads, in situ gels, nanoparticles, scaffolds, nanofibers, and nanosponges [35,36]. Several research reports (Table 2) focusing on chitosan blends with other polymers for the enhancement of drug properties such as the controlled and sustained release of drugs and improving mechanical and physical properties of drugs are discussed in the section below. Researchers have formulated floating matrix tablets using chitosan and a hydroxypropyl methylcellulose (HPMC) polymer blend, incorporating furosemide. The invitro drug release of furosemide was enhanced following a non-Fickian diffusion mechanism. The results concluded that the formulation followed zero-order kinetics with a flotation period of more than 8 h and was suitable for the oral control release of furosemide [37]. Investigators formulated and evaluated pH-sensitive controlled release microspheres. The microspheres were prepared by blending chitosan and acrylamide-grafted polyethylene glycol and incorporating cefixime using the crosslinking and precipitation methods. FTIR spectroscopy has revealed that there is no interaction between the drug and the polymer, while 80% of the drug was released from the microspheres and followed the Higuchi release kinetics [24]. Chitosan and HPMC-blended microspheres encapsulating fluorouracil were prepared using the water-in-oil emulsion technique. The formulation was evaluated through FTIR spectroscopy, which confirmed the crosslinking reaction; scanning electron microscopy (SEM) revealed the spherical shape of particles; and differential scanning calorimetry (DSC) and X-RD revealed the uniform distribution of drug particles in the formulation [38]. Cornstarch was blended with chitosan through a melt extrusion method. FTIR spectroscopy and X-ray diffraction (XRD) studies have revealed the possible interactions between chitosan and the starch molecules. The incorporation of thermoplastic chitosan was shown to cause a decrease in the tensile strength and stiffness of the films [39]. Raza et al., created carboxymethyl chitosan and poly(vinylpyrrolidone) based on hydrogels using electron beam irradiation with different dose variations for drug delivery applications. The hydrogel was characterized by its swelling index, chemical, cell cytotoxicity, thermal, and drug release properties. FTIR studies confirmed the absence of drug–polymer interactions, and SEM studies confirmed the porous structure. Cell cytotoxicity analysis indicated good cell viability in cell line studies. More than 90% of the drug release was observed within 168 h. Thus, this carrier shows promising results as a sustained drug delivery system [40]. Tetracycline hydrochloride was loaded into nanofibrous membranes prepared bypoly(-pentadecalactone-co-caprolactone)/gelatin/chitosan for the treatment of skin infections. Up to 96.5% of the drug release was observed within 14 days, with an initial burst release of 11.8%, which demonstrated good antibacterial activity against *Staphylococcus aureus* and *Bacillus subtilis* [41]. Chitosan- and tamarind-crosslinked poly(methacrylic acid) hydrogels for a pH-responsive delivery were generated using an aqueous free radical polymerization approach. The hydrogels showed an entrapment efficiency of 54–108% with a swelling index of 38.67. It showed 94.51% drug release and followed zero-order kinetics. Thus, it was concluded that the hydrogel-based system is an excellent drug delivery approach when controlled drug delivery is desired [42]. Researchers created chitosan and cyclodextrin nanofibers loaded with triclosan for a prolonged and sustained antibacterial effect [43]. Researchers prepared a pH-responsive carboxymethyl chitosan/sodium alginate-blended hydrogel loading with carvacrol. The hydrogel was characterized by its thermostability and swelling index. An increase in thermostability was observed with the increase in chitosan. The swelling index increased with the increase in pH of the buffer solution. The drug release kinetics showed a Fickian diffusion mechanism. It had good biocompatibility and biosafety and presented a system of precise drug release and intelligent preservation [44].

Researchers have undertaken a study to enhance the mechanical and physical properties of chitosan. In this study, chitosan was blended to formulate films. The blended films exhibited the amorphous nature of films and, therefore, could be used for the adsorption process [45]. In another study, the authors have prepared chitosan blends with the use of polyvinyl alcohol (PVA), and the addition of formaldehyde, thereby enhancing the thermal stability of the blends. The presence of formaldehyde decreased the solubility due to the aldehyde (-CHO-) group in formaldehyde, thereby allowing for the formation of crosslinks with the amine (-NH_2_-) group of chitosan [46]. Moreover, chitosan blends with CMC that were prepared with the addition of glycerol have displayed an enhanced thermal stability. When CMC crosslinks with formaldehyde, it enhances the blend’s thermal stability as compared to pure CMC [47]. Researchers have prepared chitosan-blended membranes with collagen by using the solvent evaporation method. These membranes were used in wound dressings and were evaluated for tensile and elongation strength by using infrared spectroscopy. The -CH, -OH, -COC, and -NH group’s intensities were found to be reduced in chitosan membranes, whereas the collagen membranes revealed the presence of amide I, II, and III groups [48]. Li, in 2008, blended chitosan/PEO films in order to inhibit food-borne pathogens and as an absorbent for heavy metals [49]. Hydroxyethylacryl chitosan and PVA blended films have been prepared through the casting technique, and the tensile properties of the blended films have been studied. The tensile strength and the elongation increased as the amount of PVA were increased. The prepared formulation was found to be more thermally stable than PVA, and the biomaterials were found to be non-toxic and did not produce any chemicals injury to living cells in an indirect cytotoxicity test using human bone sarcoma cells [50]. Tocosomes were prepared by blending chitosan and poly(N-isopropylacrylamide) and then coating them with sunitinib. The composition was synthesized using a Mozafari and robust method. The developed tocosomes were found to have a lower critical solution temperature greater than 37 °C and are suitable for hyperthermia and the spatio-temporal release of drug particles [51]. The researchers examined the behavior of a novel nanoparticles-in-nanofibers delivery system made of alginate nanoparticles that were loaded with capsaicin and implanted into nanofiber mats made of polycaprolactone and chitosan. The nanofiber mats were prepared using the electrospinning method, and the alginate nanoparticles were prepared using nanoemulsion templates. The nanocarrier showed effective inhibition ofMCF-7 human breast cells and showed non-toxicity against human dermal fibroblasts. Thus, the prepared nanocarriers show potential for the long-term and controlled release of capsaicin for the prevention and management of cancer [52]. To distribute bupivacaine for prolonged anesthesia and pain relief, the study aimed to develop chitosan with genipin hydrogels as hydrophilic lipid shell-loaded poly(-caprolactone) nanocapsules. SEM, FTIR, and XRD studies support the inclusion of poly(-caprolactone) nanocapsules in chitosan-geneipin hydrogels. No cytotoxicity was observed when evaluating cellular viability. The hydrogel improved the skin permeation of the drug by 3–5-fold in 24 h with a prolonged release of the drug [53]. Researchers explored the sustainability of the drug release, and 5-fluorouracil and Fe_3_O_4_ nanoparticles were enclosed in core–shell polycaprolactone/chitosan nanofibers. The nanofibers had a drug loading efficiency of 65–86%. It is recommended to use this core–shell drug release system as pot-surgical implants for various cancer therapies, such as liver or colorectal cancer [54]. As a control group, a polymer mixture made of PVA and chitosan was electrospun into nanofibrous mats. The results showed that the nanofibrous mats promoted collagen matrix deposition and re-epithelization, accelerated angiogenesis, and increased wound closure rates at all stages of wound healing. The results of the current study showed that nanofibrous mats could successfully reduce the time required for wound healing by suppressing inflammatory activity, thereby making them viable candidates for the treatment of diabetic wounds that are difficult to heal [55]. Various nanocarriers incorporating anti-cancerous drug were synthesized for ablation of cancerous cells with a controlled release of drug such as methotrexate [56] and doxorubicin [57]. In one of the recent studies, a researcher modified chitosan hydrogel with lanthanum and incorporated epigallocatechin gallate. The hydrogel showed the controlled release of the drug in simulated body fluids, showing antifungal properties [58]. Alginate, carboxymethyl chitosan, and aminated chitosan were employed to formulate polyelectrolyte complexes microcapsules for an effective release of diclofenac sodium. Increasing the concentration of aminated chitosan prevented the burst release of the drug and initiated a controlled drug release that was pH simulated [59].

## 3. Applications of Chitosan Blends in Drug Delivery

**Figure 4:**
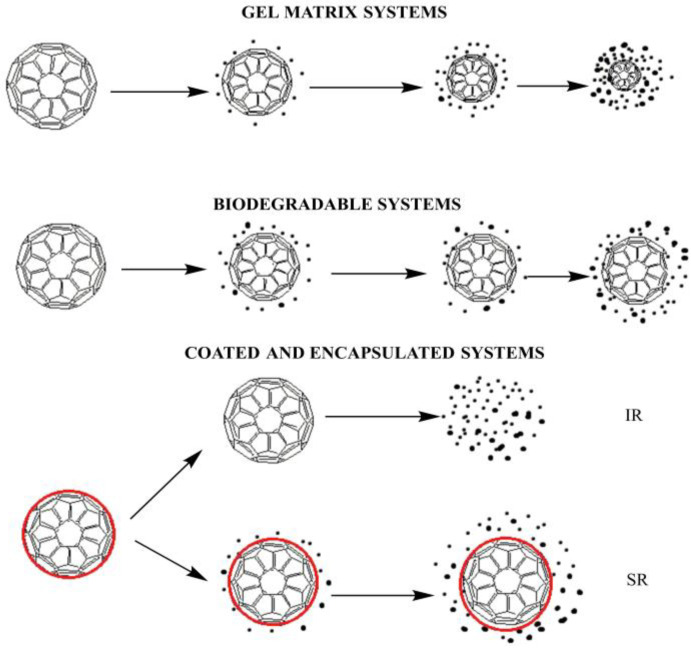
Disintegration of drug in various systems; IR, immediate release; SR, sustained release.

Depending on the type of delivery method, the drug release from polymer-based dosage forms might involve different mechanisms of action. The polymer is uniformly mixed with a drug in a gel matrix system or in biodegradable systems. The diffusion of the drug through the matrix and the gel erosion or biodegradation results in drug release. A polymer produces a film or a shell over the medication particles in a coated or an encapsulated system [60]. The film dissolves quickly in coated immediate release (IR) systems, but the drug release is gradual in the coated sustained release systems (SR) due to the diffusion taking place through an insoluble polymer film. Briefly, the mechanism of drug release from polymer blends is summarized in Figure 4 [61]. In the latter, drug molecules are represented by spheres, while the polymer is uniformly mixed with the medicine in the matrix and the biodegradable systems illustrated. The diffusion through the matrix and the gel erosion (e.g., hypromellose-based tablets) or biodegradation (e.g., poly(lactide-coglycolide)-based systems) is used to release the drug. A polymer produces a film or shell over the drug particles in the coated or encapsulated systems [62]. The film dissolves quickly in the coated IR systems, but the drug release is gradual in the coated SR systems due to the diffusion taking place through an insoluble polymer layer. This section presents the latest biomedical approaches for chitosan-based drug deliveries (e.g., oral, transdermal, topical, buccal, etc.) that are briefly described in Table 3.

### 3.1. Smart Drug Delivery Matrices

Smart drug delivery systems refer to a procedure in which medicines are not released until they reach their target tissues/organs and are only released at the specific sites of action. The smart nanotech-based delivery systems allow for an active compound to be released at specified tissues during the systemic delivery. Smart polymers are becoming essential and advantageous in the disciplines of controlled drug delivery, tissue engineering, and medical applications [63]. Various research reports were studied and are discussed in this section. Researchers have prepared chitosan/guar gum/polyvinyl pyrrolidone pH-responsive hydrogels. The pH-responsive behavior of the hydrogels has allowed for these hydrogels to be applicable for smart drug release applications [64]. Chitosan and poly(allylamine hydrochloride) blend films have also been developed as smart drug delivery matrices. A sustained drug release has been observed in phosphate buffer solution, due to the poor stability of the films in the latter. These findings demonstrate the excellent potential of blended films in providing an SR for hydrophilic or unstable drugs [65]. In another study, the developed chitosan and polyvinyl pyrrolidone blended hydrogels were found suitable to be used as controlled drug delivery systems and as smart drug delivery matrices [66]. Researchers have also investigated the chemical–physical properties of cotton cellulose nanofiber nanocomposites as well as their cytocompatibility with human embryonic kidney cells. The formulation was found to be effective in enhancing the surface roughness of the chitosan film [67]. When researchers incorporated chitosan/corn starch blends with hydroxyurea by using potassium persulfate as a smart material for the development of drug delivery systems, they found that the prepared formulation was suitable for medical applications [68]. In another study, researchers conducted an experiment on novel pH-responsive hydrogels incorporating neomycin sulfate with silane by blending chitosan and alginate with PVA for injectable controlled drug delivery in biomedical applications. The resulting hydrogel exhibited the highest swelling at an acidic pH and a low swelling at a basic and neutral pH. Moreover, it exhibited a drug release in 30 min. Thus, it could be said that these hydrogels can be used as smart, intelligent materials for an injectable controlled drug delivery [69]. The current study created indomethacin-incorporated, cyclodextrin-grafted chitosan nanofibers using the electrospinning method. The preparation of the graft was confirmed by FTIR spectroscopy, nuclear magnetic resonance, and XRD analysis. The nanofibers were found to be at the nanoscale, as confirmed by SEM, and they improved cell proliferation because of their size. The controlled release of the drug was observed in phosphate buffer solution, and it was concluded that it was appropriate for smart drug delivery systems for wound healing and tissue engineering approaches in orthopedic applications [70]. Various other research reports were compiled on smart drug delivery-based systems using drugs such as ciprofloxacin [71], mesalamine [72], Dasatinib [73], and doxorubicin [74].

### 3.2. Oral Drug Delivery Systems

Nanoparticles are employed as oral delivery vehicles for polynucleotides, macromolecules, and proteins due to their benefits of microparticle size, vast surface area, and a possibly modifiable surface that can inhibit the enzymatic degradation of the medications in the gastrointestinal tract. Due to its mucoadhesive characteristics and short opening of the mucosal cell membrane’s tight junctions, chitosan promotes absorption. In addition, the interaction between chitosan (positively charged) and mucin (negatively charged) increases the amount of time the drug is in touch with the absorptive surface. Moreover, due to its mucoadhesion, chitosan also increases the half-time of the drug’s clearance [75]. In this section, authors have compiled the research work performed on chitosan blends with other polymers toward oral drug delivery or for sustained drug release.

Researchers have prepared chitosan-poly-ε-caprolactone nanoparticles for a protein/antigen delivery system with an orally administered site-specific oral drug delivery device that was developed [76]. A chitosan-blended formulation has also been loaded into a mini pellet system. The success of the produced system was demonstrated in both in vitro and in vivo tests, with oral drug delivery giving a higher control of salmon calcitonin release when compared to the commercial product [77]. In another study, gingerol/soya lecithin were blended to develop a phytosome by using the anti-solvent precipitation method. The prepared formulation was characterized for %yield, %entrapment efficiency, particle size, drug loading, and physical compatibility. The formulation was found to occur within the nano-range, while its drug loading and encapsulation efficiency was found to be 86 and 8%, respectively. Moreover, the formulation displayed a potent antioxidant, antibacterial, and anti-inflammatory activity. It also enhanced the bioavailability of the drug, showed a better SR profile, and prolonged the oral absorption of the drug [78]. In a different study, alginate/N-succinyl-chitosan-blended microspheres were developed, and the formulation appeared to be a good candidate for inflammatory bowel diseases treatment [79]. Researchers that have attempted to develop a microflora-triggered colon-targeted drug delivery system based on polysaccharides have prepared tablets that were coated with Eudragit RLPO/chitosan blends. In their study, first-order release kinetics were observed, followed by a Higuchi spherical matrix release. In vivo studies showed a delayed T_max_, an increased absorption time, as well as a decreased C_max_ and absorption rate constant, thereby certifying a reduced systemic drug toxicity [80]. Apart from the multiparticulate systems for oral drug delivery, chitosan with a suitable blend of polymers can be prepared in tablet form. Usually, chitosan is prepared in salt form for better solubility and compactability. Other polymers were blended with chitosan to modify the drug release profile for a specific purpose, e.g., combinations with ethylcellulose delay drug dissolution for colonic delivery purposes [81]. An additional colonic drug release trigger for chitosan is an enzyme produced by the microorganisms of the colon. By combining the appropriate amount and molecular weight of HMPC with the chitosan, the model drug could be delivered by a mechanism that involves pH, time, and an enzyme [82]. Investigators developed novel elastic films based on chitosan and poly(3-hydroxypropyl ethyleneimine) for the delivery of haloperidol. The blend was fabricated to increase the mechanical properties for effective delivery of haloperidol. The films were prepared using both the casting and evaporation methods. The film solubility was found to be 1.5%, and the film allowed for rapid drug release within 30 min, after which the films disintegrated, demonstrating the formulations are suitable for application to the mucosal surfaces. The formulated films were considered to be promising carriers for the delivery of haloperidol [83].

### 3.3. Mucoadhesive Drug Delivery System

Mucoadhesive drug delivery systems have sparked a lot of interest due to their ability to prolong the residence time of dosage forms at absorption sites or at the site of action. As a result, mucoadhesive drug delivery systems allow for an SR of loaded drug candidates with minimal drug degradation. Various natural, synthetic, and semi-synthetic polymers have been used to generate diverse mucoadhesive drug delivery methods. Natural polymers (such as chitosan) have been extensively used in the development of several types of mucoadhesive drug delivery systems, with efficient treatment applications [84]. In this section, the recent literature on chitosan blends with other polymers for buccal or mucoadhesive drug delivery is discussed along with the enhanced properties of the polymers involved.

Pectin/chitosan nanoparticles were prepared by loading meclizine and by incorporating into buccal film. The formulation possessed a nano-range structure with 90% entrapment efficiency and enhanced permeation. From the obtained results, it could be concluded that the formulation can be a favorable delivery system for curing the distressing symptoms associated with cancer chemotherapy [85]. In situ thermosensitive electro-responsive mucoadhesive gel loaded with nanocomposites has also been prepared. The nanocomposites were prepared by blending chitosan, pluronic-F127, polyaniline, and HPMC loaded with carmustine nano-co-Plex. The developed formulation may be useful for a nose-to-brain controlled drug delivery [86]. In another study, composite sponges were prepared by blending chitosan and HPMC and lyophilizing them. The formulation exhibited an increased mucoadhesion time to 6 h in 10 min. Moreover, stability studies have shown that the sponges can be kept in low humidity conditions while maintaining their soft texture and mucoadhesive properties for a month [87]. In a study reporting on betamethasone-17-valerate mucoadhesive membranes prepared for the treatment of recurrent aphthous stomatitis, the membranes were prepared by blending chitosan and polyvinylpyrrolidone through the solvent evaporation technique. The membranes exhibited an increased tensile strength and an enhanced mucoadhesion. The formulated mucoadhesive membranes were found to be a promising system for drug delivery as part of a treatment for recurrent aphthous stomatitis [88]. Finally, microspheres have been prepared by blending chitosan/pluronic F127, and by incorporating puerarin through emulsification. The blended microspheres exhibited better physical stability and increased swelling capacity, while the in vitro drug release was found to be reduced due to the improved compatibility of the drug and the matrix used. The study concluded that the formulation exhibited a good anti-protein absorption in solution [89]. Chitosan- and hypromellose phthalate-based solid dispersions were prepared using zidovudine for mucoadhesive drug delivery. The prepared solid dispersions were less than 100 nm in size and had a high drug content. No drug–polymer interactions were observed using FTIR studies. Thus, the prepared solid dispersion proved to be a feasible strategy to modulate the physio–chemical, mucoadhesive, and drug release properties [90].

### 3.4. Transdermal and Topical Drug Delivery System

Transdermal administration is a new trend allowing us to persistently administer medicines with low bioavailability and a strong first pass effect to maintain an optimal therapeutic concentration. The application of active pharmaceutical ingredients to the skin allows for a local activity and the delivery of a drug into the systemic circulation. This drug administration route has a number of advantages such as the achievement of consistent drug plasma concentrations, the removal of the hepatic first pass, a great patient compliance, and the avoidance of gastrointestinal degradation [91,92]. Various approaches to transdermal drug administration have been reported in the literature, ranging from paths to gels and polymer nanoparticles loaded with the drug. In this section, we present the research work performed on chitosan blends with other polymers for the achievement of transdermal and topical drug delivery. Researchers have prepared chitosan and HPMC blends, with and without the incorporation of propranolol HCl, through a solvent casting method. The blend revealed good film forming property and could be used in a transdermal drug delivery system [30]. In another study, investigators fabricated PVA and chitosan-blended hydrogels for the transdermal drug delivery of insulin. The nano-insulin-loaded hydrogels were characterized through SEM, FTIR spectroscopy, energy dispersive spectrometry, DSC, XRD, thermogravimetric analysis, and its mechanical properties. The results showed good compatibility, as well as good mechanical and thermal properties. Moreover, Fick’s first law of diffusion was seen in the drug release kinetics. Based on these findings, the nano-insulin loaded hydrogel appeared to be a viable non-invasive TDD device for the treatment of diabetes [93]. Researchers prepared composite films from a mixture of chitosan and gelatin, by using acetic acid with and without theophylline. Weak interactions were observed between the polymers as well as between the polymers and the drug in the casted film. SEM studies indicated a uniform dispersion of the drug throughout the polymer blend, and drug crystals were seen at higher magnifications. Hence, the prepared formulation can be used as a transdermal carrier for the systemic delivery of drugs through the skin [94]. Investigators focused on the development and characterization of HPMC/chitosan blends incorporating *Zingiber cassumunar* Roxb. In vitro release studies and skin permeation studies were conducted on these patches, and the homogenous smooth and compact patches indicated a compatibility of the mixed materials. The formulation was found to be released in a controlled manner and to permeate the skin through blended patches developed from chitosan/HPMC blends. As a result, the blended patches could be used to dispense herbal medicine [95]. Chitosan/cellulose laurate/poly(N-isopropylacrylamide-co-acrylic acid) has been blended for the fabrication of nanoparticles suitable for transdermal drug delivery systems. The nanoparticles were prepared by a microfluidic technique and were found to be in nano-range. The thermal stability of the drug-loaded nanoparticles generated using the microfluidic approach at 45 °C for one month exhibited a reduction in degradation. In vivo tests carried out on Wistar rats revealed that the use of microfluidly-generated nanoparticles can result in decreased superficial erythema and appropriate transdermal permeation. Finally, these chitosan-based nanoparticles have demonstrated a potential for being employed in transdermal multidrug administration [96]. Enrofloxacin was incorporated in chitosan and PVA-blended films by the casting and drying method for local delivery. The films were characterized by morphology, thermal behavior, crosslinking degree, thickness, drug release, chemical structure, tensile strength, weight uniformity, and swelling. The in vitro drug release showed an SR of the drug following the Higuchi release order. Thus, it could be concluded that the films are potential candidates for the use in a transdermal drug delivery system [97]. Poly(1-vinylpyrrolidone-co-vinyl acetate)/chitosan-blended films were fabricated by the solvent casting method, while the films were characterized by FTIR spectroscopy, UV-visible, thermal analysis, and water contact angle. The formulation showed high thermal stability and glass transition temperature. The swelling property of formulation was enhanced. The blended films exhibited potential in terms of ultraviolet screening barriers, wound dressing material, and drug delivery [98]. Researchers developed films with the blend of chitosan and polyhydroxybutyrate for wound infection. The film has shown high breathability, mechanical strength, and thermal stability. From the undertaken XRD and FTIR spectroscopy studies, the authors concluded that the interaction between the drug and the polymers resulted in reduced film crystallinity. In fact, a porous morphology was observed through SEM. The blend revealed 96.6% encapsulation efficiency and an enhanced SR profile of the drug for 48 h. Moreover, within 20 min, the fabricated blend had an excellent blood clotting potential [99]. Semi-interpenetrating chitosan/PEG sponges have been designed by crosslinking PEG in the chitosan matrix through a nucleophilic thiol-ene addition reaction. FTIR analysis of the molecular structure of the developed microsponges has revealed an intermolecular interaction between PEG and chitosan, as well as the presence of a crosslinked PEG network in the chitosan matrix. Other improved properties like higher Young’s modulus (mechanical strength) and the stability at physiological pH, opened the way for the application of this biomaterial in topical drug delivery [100]. On the other hand, gel formulations using a blend of chitosan, PVA, and gellan gum (Gelrite™) for a sustained ocular delivery of besifloxacin (BSF) have been prepared. The results have shown that the BSF sol-gel system was sensitive enough and, therefore, underwent instantaneous phase transition upon getting physiological stimulation. The ex vivo permeation experiments indicated that the developed formulation was able to enhance the retention of BSF at the corneal surface. The results of the undertaken gamma scintigraphy also revealed that a higher concentration of drug is retained at the corneal surface. In addition, the optimized BSF sol-gel system has exhibited an enhanced antibacterial activity when compared to the BSF suspension [101]. *Periplaneta americana* extract (PAE) films that were prepared by blending PVA, hydroxypropyl chitosan, and carbomer with glycerol as a plasticizer, exhibited pro-fibrogenic and pro-angiogenic effects, as well as enhanced swelling ability. SEM and FTIR spectroscopy revealed excellent wound healing properties. This newly discovered hydrogel film-loading PAE would be useful for acute cutaneous wound health care [102]. Finally, researchers have also prepared vancomycin-loaded polyethylene oxide/chitosan-blended nanofibers by employing the electrospinning method. The prepared formulation has a potential to promote wound healing by reducing the side effects of vancomycin as a topical antimicrobial agent [103]. Ali et al. developed a methylcellulose- and chitosan-based bio-nanocomposite transdermal patch using the solvent evaporation method. The prepared patches showed in vitro cytocompatibility, and a sustained drug release was observed along with enhanced bioavailability. Thus, the patches have a broad prospect for transdermal drug delivery [104]. Chitosan, PVA, and polyethylene glycol were blended and evaluated for use in transdermal patches using tramadol as a model drug. The researchers found no gaps or cracks in prepared patches, as confirmed by SEM studies. The patches showed a good permeation rate and are suggested to be excellent candidates for transdermal drug delivery [105].

### 3.5. Intranasal Drug Delivery Systems

The systemic availability of medications only available through intravenous administration has been extended to include nasal drug administration. The wide surface area, permeable endothelium membrane, high total blood flow, avoidance of first pass metabolism, and easy accessibility are the advantages of opting for nasal drug delivery systems. Numerous chemicals, peptide, and protein medications have been administered nasally for systemic treatment in recent years and are discussed below in this section. After drug administration, the drug is rapidly removed from the nasal cavity, leading to quick systemic drug absorption. Drugs delivered intranasally can bypass the blood–brain barrier and undergo minimal hepatic and intestinal metabolism, thereby allowing them to enter the brain directly. This method has consistently been proven to be both economical and efficient. Drugs are delivered to the brain via olfactory, neuronal, and trigeminal routes by using several innovative formulations [106]. On the other hand, a new formulation containing chitosan, gelatin, and a chitosan quaternary ammonium salt has been developed and tested as a viable carrier for the intranasal delivery of insulin. The hydrogel was characterized for various evaluating parameters, the optimized thermosensitive hydrogel was blended with insulin in a clinical formulation and dosage, and the insulin release procedure was examined by using electrochemiluminescence technology [107]. The optimized formulation showed low gelation time, uniform pore structure, and a desirable swelling rate, which resulted in adequate encapsulation and a prolonged release of insulin for 24 h.

**Table 3:** Studies reporting the use of chitosan blends in various drug delivery systems.

Chitosan Blend	Nano Carriers	Drug Delivery System	Outcomes	Ref.
Chitosan and HPMC	Film	Transdermal	The blend exhibited a good forming property	[30]
Chitosan/guar gum/polyvinyl pyrrolidone	Films	Smart	pH-responsive behavior of the hydrogels; these hydrogels are suitable for drug release applications	[64]
Chitosan-poly(allylamine hydrochloride)	Films	Smart	Results revealed an excellent potential of blend films as part of sustained-release drug delivery systems for hydrophilic or unstable drugs	[65]
Chitosan and polyvinyl pyrrolidone	Hydrogels	Smart	Fabricated hydrogels can be used as controlled drug delivery systems	[66]
Cotton cellulose nanofiber (CCN)/chitosan nanocomposites	Nano composite	Smart	It could be a promising biocompatible biomaterial for a number of biomedical applications	[67]
Chitosan/corn starch	Composite films	Smart	Results concluded that the corn starch/chitosan smart materials may be suitable for medical applications such as drug delivery systems to a rhabdomyosarcoma (RD) cell line, as confirmed by the availability and the morphology of the RD cell line	[68]
Chitosan and alginate	Hydrogels	Smart	Hydrogels can be used as smart intelligent materials for injectable controlled drug delivery for biomedical applications	[69]
Β-CD grafted chitosan	Nanofibers	Smart	The formulated nanofibers could be preferred for orthopedic diseases	[70]
Carboxylated graphene oxide, alginate beads coated with aminated chitosan	Microbeads	Smart	The microbeads were viable to human cell (98%) and can be applicable for oral drug delivery of antibiotics	[71]
Aluminosilicate nanoparticles, curcumin, and chitosan	Nanocomposites	Smart	The drug showed high efficacy performance up to 90% for 90 h.	[72]
Chitosan, 3,3-dithiodipropionic anhydride	Micelles	Smart	The formulated micelles were effective against inflammatory diseases	[73]
Glutamic acid and chitosan	Beads	Smart	The beads showed a promising use in a controlled delivery of anticancer agents	[74]
Chitosan-poly-ε-Caprolactone	Nanoparticles	Oral	Displayed an advantageous release profile for oral delivery	[76]
Chitosan-Polystone^®^ M.	Mini pellet system	Oral	The prepared system provided a greater control of release of salmon calcitonin	[77]
Chitosan/soya lecithin	Nano particles	Oral	Displayed a significantly sustained release profile, with effective antibacterial activity	[78]
Chitosan/Eudragit RLPO	Tablet	Oral	The formulation showed a delayed T(max), a prolonged absorption time, a decreased C(max), and an absorption rate constant (Ka) indicating a reduced systemic toxicity of the drug	[80]
Chitosan/ethylcellulose	Tablet	Oral	A dual time- and pH-control control strategy for colonic drug delivery	[81]
Chitosan/HPMC	Tablet	Oral	Delivery of 5-aminosalicylic acid to the colon using pH-, time-, and enzyme-dependent triggers	[82]
Chitosan/poly (3-hydroxypropyl ethyleneimine)	Films	Buccal	The blended film showed better mechanical strength than non-blended chitosan, and rapid drug release was observed	[83]
Pectin/chitosan	Nanoparticles	Mucoadhesive	The formulation can be a favorable delivery system for curing distressing symptoms associated with cancer chemotherapy	[85]
Chitosan/polyaniline and HPMC	Nanocomposites	Mucoadhesive	The developed formulation may be useful for a nose-to-brain controlled drug delivery	[86]
Chitosan and HPMC	Composite sponges	Mucoadhesive	The formulation allowed for the sponges to be kept in low humidity conditions while maintaining their soft texture and mucoadhesive properties for one month	[87]
Chitosan and polyvinylpyrrolidone	Mucoadhesive membranes	Mucoadhesive	Mucoadhesive membranes were shown to be a promising system for the delivery of drugs used as a treatment of recurrent aphthous stomatitis	[88]
Chitosan/pluronic F127	Microspheres	Mucoadhesive	The formulation exhibited a good anti-protein absorption in solution	[89]
PVA and chitosan	Hydrogels	Transdermal	The results revealed a good compatibility, as well as good mechanical and thermal properties	[93]
Chitosan and gelatin	Film	Transdermal	The prepared formulation can be used as a transdermal carrier for the systemic delivery drugs through the skin	[94]
HPMC/chitosan	Patches	Transdermal	The blended patches might be used to dispense herbal medicine	[95]
Chitosan/cellulose laurate/poly(N-isopropylacrylamide-co-acrylic acid)	Nanoparticles	Transdermal	These chitosan-based nanoparticles could be employed for the delivery of a transdermal multidrug administration	[96]
Chitosan and PVA	Films	Transdermal	Drug release showed a sustained release of the drug following the Higuchi release order, and the blend could be used in a transdermal drug delivery system	[97]
Poly(1-vinylpyrrolidone-co-vinyl acetate)/chitosan	Films	Transdermal	The formulation exhibited high thermal stability and glass transition temperature; the swelling property of the formulation was found enhanced	[98]
Chitosan and polyhydroxybutyrate	Films	Transdermal	The film exhibited high breathability, mechanical strength, and thermal stability	[99]
Chitosan/PEG	Sponges	Topical	The formulation exhibited a higher Young’s modulus (mechanical strength) and stability at physiological pH	[100]
Chitosan, PVA	Gel	Topical	The study revealed that a higher concentration of drug is retained at the corneal surface	[101]
PVA, hydroxypropyl chitosan	Hydrogel films	Topical	This newly discovered hydrogel film-loading PAE would be useful for acute cutaneous wound health care	[102]
Polyethylene oxide/chitosan	Nanofibers	Topical	The formulation has a potential to promote wound healing	[103]
Chitosan, polyvinyl alcohol and polyethylene glycol	Patches	Transdermal	The patches showed good permeation rates and showed no gaps or cracks in patches	[105]
Chitosan/gelatin	Hydrogel	Intranasal	The formulation can deliver insulin to the bloodstream via intranasal administration	[108]

## 4. Compatibility of Polymer Blends

The ability of individual component substances to exhibit interfacial adhesion in either an immiscible polymer blend or a polymer composite (in which the interfaces between phases or components are maintained by intermolecular forces, chain entanglements, or both, across the interfaces) is referred to as “compatibility” [108]. The degree of compatibility is mostly determined by the interaction between the polymeric phases of the polyblends. The properties of a heterogeneous polymer blend are determined by the compatibility between the polymer phases. By altering the interaction forces between the constituents, one can obtain materials with the desired mechanical properties [109,110]. The interfacial tension defines the contact between the polymer phases of a polymer system, and when it approaches zero, the blend becomes miscible. In other words, if the phases interact strongly, the polymer blend will be miscible [111]. Large interfacial tensions can cause phase separation, with the separated particles possibly coalescing, thereby resulting in an increase in particle size and a reduction in terms of the mechanical characteristics. The inclusion of interfacial chemicals known as “compatibilizers” can help minimize interfacial tension [7]. Compatibilizers are molecules with hydrophobic and hydrophilic regions that can be positioned along the interface between the two polymer phases, thereby lowering the interfacial tension and increasing the polymer blend compatibility. Compatibility leads to smaller dispersed particles, better phase stability, and improved mechanical properties [112,113]. Techniques such as thermogravimetric analysis, universal testing machines, and dynamic mechanical thermal analysis can be used to characterize the physical properties of compatible, incompatible, and miscible blends [114].

## 5. Clinical Trials

Ongoing and completed clinical trials have been compiled in Table 4; these trials assess the SR of drug or other applications (such as wound healing, etc.) of chitosan-based polymer blends.

## 6. Future Perspectives

Chitosan blends have shown promising results as drug delivery systems due to their compatibility, biodegradability, and mucoadhesive properties. In the future, chitosan blends are expected to play a significant role in drug delivery due to their versatility and ability to be tailored for specific applications such as targeted drug delivery, controlled release, and combination therapy. As discussed in the review, intranasal drug delivery systems seem to be effective in targeting the brain, and there is a great need for researchers to explore intranasal drug delivery. Chitosan blend-based nanocarriers can effectively distribute drugs to specific areas by keeping them locally and by allowing for a longer period of absorption. Chitosan’s mucoadhesion and absorption improvement allows for medications to be delivered straight from the nose to the brain [120]. Similarly, chitosan nanoparticles can successfully address lung infections and colon disorders on a local level [121]. A chitosan-based nasal formulation of morphine (Rylomine^TM^) is now undergoing Phase 2 clinical trials in the United Kingdom and the European Union, as well as Phase 3 clinical trials in the United States [122]. We hope that future research on chitosan nanoparticles (made from chitosan or chitosan derivatives) will include the undertaking of toxicity tests in humans. According to this review, chitosan and its blends have a promising future because of the enhanced distinctive qualities of their unique biocompatibility, biodegradability, mechanical and thermal stabilities, barrier avoidance, and non-toxicity, which suggests their uniqueness in biomedical applications based on numerous recent publications.

## 7. Conclusions

There are numerous polymer blend combinations that are possible owing to the large number of currently available polymers with established safety profiles and histories of use in pharmaceutical and biomedical products. These combinations have the potential to address many of the formulation issues that arise during the development of new drug products. Characterizing and understanding the nature of the polymer–polymer interactions in these systems is crucial for the appropriate selection and use of these polymer blends. This allows for the creation of innovative polymer blends that can be consistently synthesized and meet the needs and requirements for polymer-based drug delivery. Chitosan appears to be an environmentally acceptable biopolymer available at a fair price. Although, chitosan possesses inherent non-toxicity, biodegradability, and biocompatibility, its mechanical and antibacterial capabilities are insufficient. As a result, chitosan should be changed or modified prior to any therapeutic use. Chitosan can be blended with starch, pectin, alginate, cellulose, and dextran in order to examine how effective chitosan blends could be useful for biological applications. Chitosan-based blends have been proven to be attractive substrates for a wide range of applications. We, herein, provide an overview of recent breakthroughs regarding chitosan-based blends. Future research on chitosan-based blends should focus on the understanding of the mechanisms involved in the observed therapeutic and antibacterial effects of these blends.

## Figures and Tables

**Table 1:** Classification of polymers.

Classification of Polymers		Description	Examples
Based on their source	Natural polymers	Natural polymers occur in nature (i.e., in plants and animals)	Proteins, cellulose, starch, rubber
	Synthetic polymers	Synthetic polymers can be artificially created or synthesized in the lab	Polyethylene or nylon fibers
	Semi-synthetic polymers	Semi-synthetic polymers can be obtained by modifying natural polymers in the lab	Vulcanized rubber, cellulose acetate
Based on their structure	Linear polymers	The monomers in these are linked together to form a long chain; these polymers have high melting points and are of higher density	Polyvinyl chloride
	Branch chain polymers	Monomers are joined together to form a long, straight chain with some branched chains of different lengths	Low-density polyethylene
	Crosslinked or network polymers	Monomers are linked together to form a 3D network; they contain strong covalent bonds and are brittle and hard	Bakelite, melamine
Based on their polymerization	Addition polymers	They are formed by the repeated addition of monomer molecules	Ethene _n_(CH_2_=CH_2_) to polyethene—(CH_2_-CH_2_)_n_-
	Condensation polymers	These are formed by the combination of monomers, with the elimination of small molecules such as water, alcohol, etc.	Hexamethylenediamine and adipic acid to produce Nylon-66 by eliminating water molecules
Based on their molecular forces	Elastomers	These are rubber-like solid polymers that are elastic in nature	Rubber bands
	Thermoplastic	These are long-chain polymers in which intermolecular forces hold the polymer chains together	Polystyrene or PVC
	Thermosetting	These are semi-fluid in nature with low molecular mass	Bakelite
	Fibers	These polymers have a thread-like structure and can easily be woven	Nylon-66

**Table 2:** Brief description of various research reports on polymer blends for controlled and sustained release of drugs.

Polymers	Carrier System	Drug	Method	Outcomes	Ref.
Chitosan and acryl amide-grafted polyethylene glycol (PEG)	Microspheres	Cefixime	Crosslinking and precipitation	It could be an effective biodegradable carrier for antibiotics	[24]
Chitosan–HPMC	Floating tablets	Furosemide		The tablets exhibited first-order kinetics with a flotation period of more than 8 h with a controlled release of drugs	[37]
Chitosan–HPMC	Microspheres	Fluorouracil	Water-in-oil emulsion techniques	Developed microspheres were effective against preventing cancer	[38]
Chitosan–cornstarch	Films	-	Melt extrusion method	Films were produced with better extensibility and thermal stability	[39]
Chitosan–polystyrene	Films	-	Stirring method	The films showed good film forming ability, biodegradability, and biocompatibility	[45]
Chitosan–PVA	Films	-	Stirring method	The film showed good thermal stability and mechanical properties	[46]
Chitosan–carboxy methyl cellulose (CMC)	Blend	-	Stirring method	The blend showed enhanced thermal stability	[47]
Chitosan–collagen	Membranes	-	Solvent evaporation method	The formulated membranes were effective for wound dressing applications	[48]
Chitosan–PVA	Films	-	Casting method	The films were effective in inhibiting food-borne pathogens and as an adsorbent	[49]
Hydroxyethylacryl–chitosan and polyvinyl alcohol	Films	-	Casting technique	The film was thermally stable and had antibacterial properties	[50]
Carboxymethyl chitosan and poly(vinylpyrrolidone)	Hydrogels	Kanamycin	Electron beam irradiation technique	The drug carrier was effective for prolonged drug release and can be used for different biomedical applications	[40]
Poly(ω-pentadecalactone-co-ε-caprolactone)/gelatin/chitosan	Nanofibrous membranes	Tetracycline hydrochloride	Electrospinning technique	The membranes could be effectively used to treat skin infections	[41]
Chitosan/tamarind crosslinked poly(methacrylic acid)	Hydrogel	Cytarabine	Aqueous free radical polymerization technique	The hydrogel was found to be a promising drug delivery device for controlled drug delivery	[42]
Chitosan–cyclodextrin	Nanofibers	Triclosan	Electrospinning method	The nanofibers showed prolonged drug release with good antibacterial properties	[43]
Carboxymethyl chitosan/sodium alginate	Hydrogels	Carvacrol	-	The hydrogel showed good biocompatibility and biosafety	[44]
Chitosan/poly(N-isopropyl acrylamide	Tocosomes	Sunitinib	Mozafari method and robust method	The tocosome indicated a lower critical solution temperature value beyond 37 °C and was suitable for hyperthermia and the spatio-temporal release of drug particles	[51]
Chitosan and polycaprolactone	Nanofibers	Capsaicin	Electrospinning method	The nanocarriers were effective against inhibiting MCF-7 human breast cells and showed no toxicity to human dermal fibroblasts	[52]
Chitosan and genipin	Hydrogels	Bupivacaine	-	The polymeric hydrogels showed a porous structure. Cellular viability showed no cytotoxicity. The hydrogel improved the skin permeation of the drug by 3–5 folds in 24 h, with a prolonged drug release	[53]
Polycaprolactone/chitosan	Nanofibers	5-fluorouracil and iron oxide nanoparticles	Electrospinning method	The blend was sustainable and could be used as postsurgical implants for various cancer treatments	[54]
Chitosan/polyvinyl alcohol	Nanofibrous mats	Pulsatilla	Electrospinning method	Nanofiber mats promoted the healing of diabetic wounds	[55]
Carboxymethyl chitosan/oxidized pullulan	Biodegradable mesoporous silica nanoparticles incorporated in hydrogel	Methotrexate	Nanoparticles were incorporated in hydrogel through Schiff base reaction	The system showed high drug loading capacity, good pH responsive triggered disintegration of drug with more than 85% of drug release	[56]
Poly(2-(diisopropylamino)teeth methacrylate and PEGylated carboxymethyl chitosan	Sheddable shell based delivery system	Doxorubicin	Solvent co-precipitation technique	The system showed high drug loading capacity, good pH responsive triggered disintegration of drug with more than 85% of drug release	[57]
Chitosan and lanthanum	Hydrogel	Epigallocatechin gallate	-	The drug was equally distributed in hydrogel showing controlled drug release with antifungal properties	[58]
Alginate, carboxymethyl chitosan and aminated chitosan	Polyelectrolyte complexes microcapsules	Diclofenac	Ionic gelation technique	Increasing the concentration of aminated chitosan, controlled drug release was achieved and could effectively deliver diclofenac sodium at site	[59]

**Table 4:** List of ongoing and completed clinical trials using chitosan-based polymer blends.

Clinical Trial No.	Title	Intervention/Treatment	Study Type/Participants	Ref.
NCT02198703	Randomized, double-blind, placebo-controlled clinical trial for evaluating the efficacy of ab-life probiotic product on the LDL-cholesterol reduction in the moderate hypercholesterolemia	Dietary supplement: probiotics bacterial strains and placebo	Interventional/104 participants	[115]
NCT03280849	Chitosan scaffold for cellular floor repair in endoscopic endonasal transsphenoidal surgery	Other: implant of bilaminar chitosan scaffold	Interventional	[116]
NCT01597817	A randomized controlled trial of the efficacy and safety of a biofunctional textile in the management of atopic dermatitis	Other: chitosan-coated and non-coated textile	Interventional	[117]
NCT03320525	A single center, open-label, Phase 1 study to evaluate the pharmacokinetic profile of T-ChOS™ in subjects with advanced solid tumors (CHITIN)	Dietary supplement: T-ChOS (Phase-1)	Interventional	[118]
NCT03712371	Study of chitosan for pharmacologic manipulation of AGE (advanced glycation end products) levels in prostate cancer Patients	Drug: chitosan (Phase-1 and-2)	Interventional	[119]

## Data Availability

Data sharing not applicable.
